# Successional Change of the Fungal Microbiome Pine Seedling Roots Inoculated With *Tricholoma matsutake*

**DOI:** 10.3389/fmicb.2020.574146

**Published:** 2020-09-25

**Authors:** Ki Hyeong Park, Seung-Yoon Oh, Shinnam Yoo, Myung Soo Park, Jonathan J. Fong, Young Woon Lim

**Affiliations:** ^1^School of Biological Sciences, Institute of Microbiology, Seoul National University, Seoul, South Korea; ^2^Department of Biology and Chemistry, Changwon National University, Changwon, South Korea; ^3^Science Unit, Lingnan University, Tuen Mun, Hong Kong

**Keywords:** fungal diversity, microbiome, network analysis, *Pinus densiflora*, *Tricholoma matsutake*, pine mushroom, ectomycorrhizal fungi

## Abstract

The pine mushroom (*Tricholoma matsutake*; Agaricales, Tricholomataceae) is an ectomycorrhizal fungus that produces a commercially valuable, edible mushrooms. Attempts to artificially cultivate *T. matsutake* has so far been unsuccessful. One method used to induce *T. matsutake* to produce fruiting bodies of in the wild is shiro (mycelial aggregations of *T. matsutake*) transplantation. *In vitro* ectomycorrhization of *T. matsutake* with seedlings of *Pinus densiflora* has been successful, but field trials showed limited production of fruiting bodies. Few studies have been done to test what happens after transplantation in the wild, whether *T. matsutake* persists on the pine seedling roots or gets replaced by other fungi. Here, we investigated the composition and the interaction of the root fungal microbiome of *P. densiflora* seedlings inoculated with *T. matsutake* over a 3 year period after field transplantation, using high-throughput sequencing. We found a decline of *T. matsutake* colonization on pine roots and succession of mycorrhizal fungi as *P. densiflora* seedlings grew. Early on, roots were colonized by fast-growing, saprotrophic Ascomycota, then later replaced by early stage ectomycorrhiza such as *Wilcoxina*. At the end, more competitive *Suillus* species dominated the host roots. Most of the major OTUs had negative or neutral correlation with *T. matsutake*, but several saprotrophic/plant pathogenic/mycoparasitic species in genera *Fusarium*, *Oidiodendron*, and *Trichoderma* had positive correlation with *T. matsutake*. Four keystone species were identified during succession; two species (*Fusarium oxysporum*, and *F. trincintum*) had a positive correlation with *T. matsutake*, while the other two had a negative correlation (*Suillus granulatus*, *Cylindrocarpon pauciseptatum*). These findings have important implications for further studies on the artificial cultivation of *T. matsutake*.

## Introduction

Ectomycorrhizal fungi are one of the most common forms of plant-fungal root symbioses in woody plants (Brundrett, 2009; Van Der Heijden et al., 2015), and improve nutrition and stress resistance of the host plant (Smith and Read, 2010; Berendsen et al., 2012; Van Der Heijden et al., 2015). Ectomycorrhizal fungi compete with each other to colonize root tips (Koide et al., 2005; Kennedy et al., 2009; Bakker et al., 2014) or co-exist (Perry et al., 1989; Yamamoto et al., 2014). Succession of the mycorrhizal community was reported in several host plants (Twieg et al., 2007). This phenomenon not only occurs in mature trees, but also in seedlings, where the dominant ectomycorrhizal taxa can change (Matsuda et al., 2009; Obase et al., 2009). Early stage ectomycorrhizal fungi (e.g., members of *Inocybe*, *Rhizopogon*, or *Suillus*) require small amount of carbon from hosts and are usually found in pine seedling in disturbed area (Colpaert et al., 1996; Sim and Eom, 2009). Arrival sequence of ectomycorrhizal fungi often influences colonization at early stages, with negative consequences for later colonizers (Alford and Wilbur, 1985; Shorrocks and Bingley, 1994). This phenomenon is called the priority effect, and has been reported in the early stage of interaction between ectomycorrhizal fungi and pine seedlings (Kennedy and Bruns, 2005; Fukumi, 2015).

The pine mushroom (*Tricholoma matsutake*; Agaricales, Tricholomataceae) produces edible fruiting bodies during symbiosis with members of Pinaceae, especially *Pinus densiflora* (Yamada et al., 2010). Due to its commercial value, artificial cultivation of *T. matsutake* has been attempted, but thus far been unsuccessful. Three unsuccessful methods to induce fruiting bodies of *T. matsutake* in the wild are inoculating cultured *T. matsutake* hyphae in soil (Lee et al., 2007), spraying of *T. matsutake* spores from fruiting body (Eto and Taniguchi, 2000), and transplanting shiro (aggregate of *T. matsutake* mycorrhiza) to uninfected pine trees (Kareki and Kawakami, 1985). The last approach of transplanting shiro to uninfected pine trees has been tried extensively in Korea (Park et al., 2007). *In vitro* ectomycorrhization of *T. matsutake* has been successful (Yamada et al., 1999, 2006; Saito et al., 2018), but field trials showed limited production of fruiting bodies (Ka et al., 2018). In order for this method to be efficient, *T. matsutake* must persist the pine seedling roots. Currently, it is unclear after pine seedlings are transplanted to the wild, *T. matsutake* persists on the pine seedling roots or gets replaced by other fungi.

Advances in high-throughput sequencing have greatly contributed to our understanding the diversity and function of fungi in various environments (Nilsson et al., 2019), and have been used to study the succession of fungal communities (Dickie et al., 2013, 2017; Voříšková et al., 2014; Hannula et al., 2017). In this study, we used high-throughput sequencing to examine the change in the root microbiome of *T. matsutake* inoculated pine seedlings after transplantation, focusing on the succession of mycorrhiza and interaction between root associated fungi. We hypothesize that (i) there is a significant change in root fungal communities during transplantation and seedling growth, and (ii) there are some fungi with positive or negative correlation with *T. matsutake* that affect the survival of *T. matsutake* on pine seedling roots.

## Materials and Methods

### Study Design and Sample Collection

This experiment was conducted at Gyeongsangbuk-do Forest Environment Research Institute in Gyeongju, South Korea. *Tricholoma matsutake* strain KBFERI 20T05 (GenBank accession no. AF367417) was cultured in K-liquid media (Park et al., 2007), and transferred to autoclaved culture vessels filled with mixed soil (perlite: peat moss = 80:1) as described by Park et al. (2007). For surface sterilization, *P. densiflora* seeds were placed in 70% ethanol for 60 s, and transferred to 2% NaClO solution for 4 min. Cleaned seeds were washed 3 times with sterile water then germinated in nutrient broth agar plates (Scharlau). Uncontaminated seedlings were transferred to culture vessels inoculated with *T. matsutake* in a sterilized culture room, then co-cultured for 3 months in a clean room illuminated with a fluorescent lamp (20°C; 25,000 lux; 24 h). Then, *P. densiflora* seedlings were moved to a greenhouse filled with autoclaved soil from a nearby pine forest. Sixteen pine seedlings were sampled at 6 different post *T. matsutake*-inoculation periods: 3 months (M03; in a sterilized culture room), 10, 17, 24, 31, and 38 months (M10, M17, M24, M31, M38; in a greenhouse). In total, 96 seedling roots were harvested.

### DNA Extraction

Harvested seedlings were placed on ice, transported to the laboratory at Seoul National University (Seoul, South Korea), and stored at−80°C prior to DNA extraction. We did a preliminary morphological examination of roots to confirm the presence of *T. matsutake* (Gill et al., 2000; Yamada et al., 2010). Seedling roots were gently washed with running water to remove debris and sterilized with 3% sodium hypochlorite for 2 min. Samples were then washed with distilled water for 5 min. Surface-sterilized roots were cut into 5 cm fragments and air-dried. For each sample, three root fragments were wet with 500 μl of cetyltrimethylammonium bromide buffer (Biosesang, Seongnam, South Korea) and ground with a mortar and pestle. For each sample, genomic DNA was extracted from seedling root using modified CTAB methods (Rogers and Bendich, 1994). We confirmed the presence of *T. matsutake* in M03 samples with *T. matsutake*-specific primers (Kim and Han, 2009).

### PCR Amplification and High Throughput Sequencing

The fungal internal transcribed spacer 2 (ITS2) region was amplified with primers ITS3 and ITS4 (White et al., 1990) with Illumina sequencing adaptors attached. PCR was conducted 3 times for each samples using AccuPower PCR PreMix kit (Bioneer, Daejeon, South Korea). PCR conditions were as follows: 94°C for 5 min, 30 cycles of 94°C for 30 s, 55°C for 30 s, and 72°C for 40 s, and 72°C for 10 min as final extension. PCR products were confirmed on 1% agarose gel (BIOFACT, Daejeon, South Korea) with gel electrophoresis. After purification using the Expin^TM^ PCR SV kit (GeneAll Biotechnology, Seoul, South Korea), a unique identifier sequence was attached to each PCR products with a second round PCR following the Nextera XT index kit protocol (Illumina, San Diego, CA, United States). Second PCR products were purified as above. Concentration of each amplicon library were measured using a NanoDrop2000 (Thermo Fisher Scientific, Waltham, MA, United States). Amplicon libraries were pooled in equimolar quantities and sequenced using Illumina MiSeq platform at Macrogen (Seoul, South Korea).

### Bioinformatics and Statistical Analysis

After sequencing, the raw data were processed using the Quantitative Insights Into Microbial Ecology v.1.8.0. (QIIME) pipeline (Caporaso et al., 2010). Fastq-join was used for merging paired-end sequences. After filtering low-quality sequences (Q < 20, length < 200 bp), 9,513,644 reads were retained for later analyses. Clustering of operational taxonomic units (OTUs) was performed with the open-source sequence search tool Vsearch v. 2.6.2 (Rognes et al., 2016) with 97% similarity level. For taxonomic identification, the most abundant sequence was selected as an OTU’s representative sequence. The UNITE v. 8.0 (Unite Community, 2019) database was used to determine OTU’s taxonomic identity with NCBI BLAST, following the criteria of Tedersoo et al. (2014). We removed chimeric sequences based on the reference database of UCHIME (Edgar et al., 2011). Singleton OTUs and non-fungal sequences were removed, and all samples were rarefied to a minimum number of sequences before further analysis. Taxonomic identity of major OTUs (OTUs with total relative abundance >0.5%) were checked manually with NCBI and UNITE databases (access date: August 26 2020). FUNGuild was used as a database for fungal trophic mode assignment (Nguyen et al., 2016).

Alpha diversity indices (Chao1 richness, Shannon’s diversity, equitability, and Good’s coverage) were calculated in QIIME. Statistical analysis was performed in R software (version 3.6.1, R Core Team, 2019). Kruskal-Wallis tests were performed to compare the diversity indices between sampling times with Dunn’s test as a *post hoc* test adjusted using the Benjamini and Hochberg method (Benjamini and Hochberg, 1995). Ordination analysis was performed by non-metric multidimensional scaling (NMDS) based on Bray-Curtis dissimilarity index using the phyloseq package (McMurdie and Holmes, 2013). Difference of community compositions among sampling times were tested with permutational multivariate analysis of variance (PERMANOVA) with 999 permutations, using the “adonis” function in the vegan package (Oksanen et al., 2018), and pairwise *post hoc* tests were done using the pairwiseAdonis package with Bonferroni correction of the Bray-Curtis dissimilarity matrix (Martinez Arbizu, 2017).

To test for correlations between species, Sparse Correlations for Compositional data (SparCC) (Friedman and Alm, 2012) network analysis was performed at the OTU level (OTUs with total relative abundance >0.5%) with the Galaxy-based analysis pipeline (Inter-Domain ecological network analysis pipeline, IDENAP, Feng et al., 2019). The significance of correlation was calculated by comparing the shuffled data from 100 permutations. Following previous studies, correlations with SparCC >0.3 and *p* < 0.05 were included (Kurtz et al., 2015). The network was visualized with Cytoscape version 3.7.2 (Shannon et al., 2003). Clusters were detected with Markov clustering algorithms (Van Dongen and Abreu-Goodger, 2012). For the overall network, species with high degree, betweenness centrality, and closeness centrality were selected as the keystone taxa. NMDS ordination and network analyses were performed without M03 samples as they were distinctly different from other samples due to high abundance of *T. matsutake* (>94% in average). Sequencing data were deposited in NCBI Sequence Read Archive (SRA) under Project ID PRJNA638021.

## Results

### Sequencing Results and Alpha Diversity Indices

**FIGURE 1:**
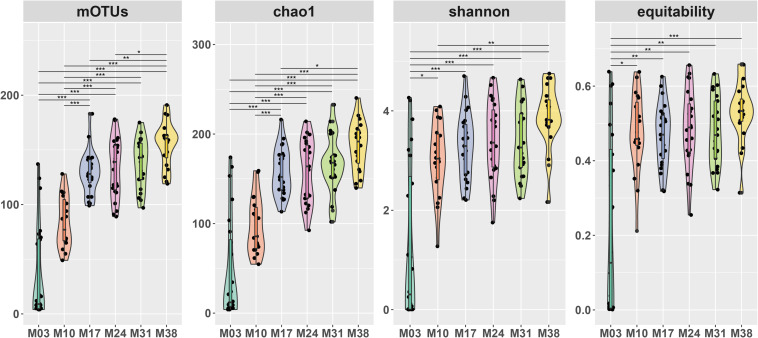
Alpha diversity of fungal communities of *Pinus densiflora* seedlings. OTUs: the number of OTUs, Chao1: Chao1 index, Diversity: Shannon’s diversity, equitability: Shannon’s equitability. The number of OTUs were calculated after rarefaction (22,000 reads). Statistically significant differences between sampling times were determined by multiple Kruskal-Wallis tests with Dunn’s test as a *post hoc* test (*p < 0.05, **p < 0.01, ***p < 0.001).

A total of 7,697,559 sequence reads were obtained from 96 samples through Illumina MiSeq sequencing with 25,244–228,456 sequence reads per sample. After rarefaction to 25,000 reads, 826 OTUs (range: 4–191) remained with a Good’s coverage of 0.998–0.999. Based on taxonomic level, the OTUs represented 8 phyla, 28 classes, 89 orders, 188 families, and 327 genera. The number of OTUs significantly increased with the age of *P. densiflora* seedlings, from 63 OTUs found in M03 (mean = 7.88 OTUs per sample) to 487 OTUs in M38 (mean = 155.88 OTUs per sample) (Figure 1). Chao1 richness, Shannon’s diversity, and equitability also showed significant increase following the growth of *P. densiflora* seedlings, especially between M03 and other sampling periods (Figure 1).

**FIGURE 2:**
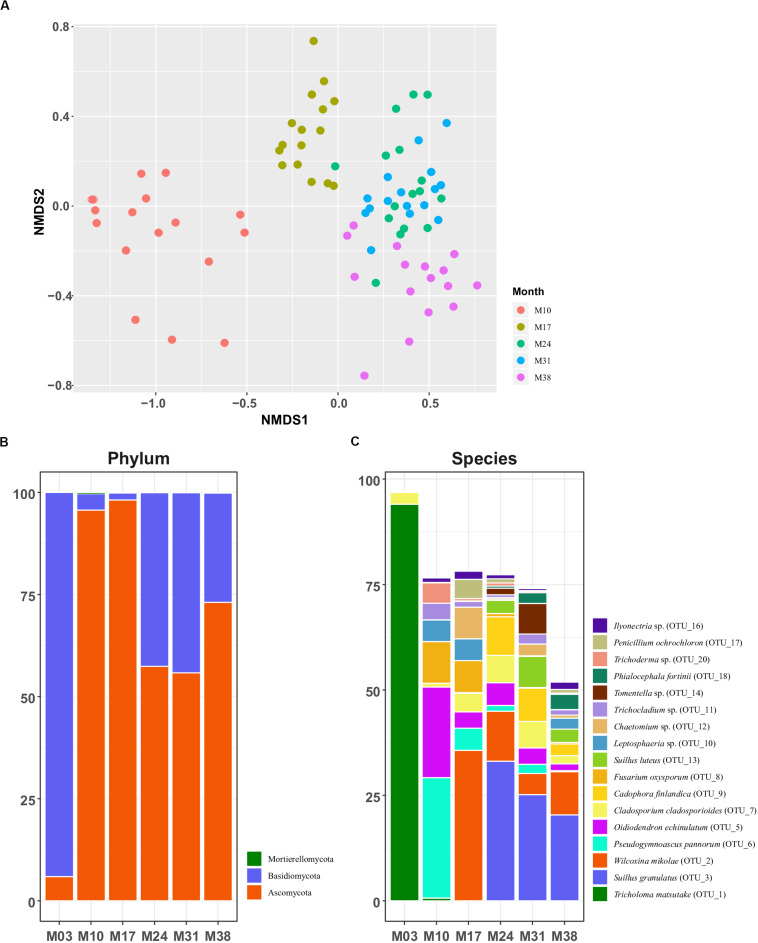
Fungal community structures of *Pinus densiflora* seedlings. **(A)** NMDS plots based on Bray-Curtis dissimilarity. Relative abundance of **(B)** major phyla and **(C)** major species. Taxa with total relative abundance higher than 1% were chosen as major taxa in **(C)**.

The NMDS ordination of Bray-Curtis dissimilarity based on OTU-level abundance revealed clear separation of fungal communities between most groups, except M24 and M31 (Figure 2A). This result was supported by pairwise adonis tests, where all but the M24-M31 comparison were statistically significant (Supplementary Table 1). We observed a significant shift of the overall fungal community in *P. densiflora* seedlings over time, based on the adonis analysis (*R*^2^ = 39.4%, *p* = 0.001; Figure 2A and Supplementary Table 1). The relative abundance of *T. matsutake* drastically decreased after the transplantation to greenhouse (M03 to M10; Figure 2C and Table 1), but *T. matsutake* was still detected in some samples (15/16 in M10; 8/16 in M17; 4/16 in M24 and M31; 2/16 in M38; Table 1).

**TABLE 1:** Average relative abundance and frequency (the number of samples with *T. matsutake*) of *Tricholoma matsutake* (OTU 1) in *Pinus densiflora* seedling roots.

Sampling groups	M03	M10	M17	M24	M31	M38
Sequence reads	376,056	2,625	44	11	5	2
Relative abundance (%)	94.014	0.65625	0.011	0.00275	0.00125	0.0005
Number of samples with *T. matsutake*	16/16	15/16	8/16	4/16	4/16	2/16

### Fungal Community Composition in *P. densiflora* Seedlings

The total abundances of major fungal phyla were relatively high: Ascomycota (64.353%) and Basidiomycota (35.516%). Abundance of the next most abundant phylum, Mortierellomycota, was low at less than 0.1% (Figure 2B). The abundance of Basidiomycota was high during the inoculation stage (M03, 94.1%), but drastically decreased after transplantation (4.0% in M10; 1.7% in M17), being replaced by Ascomycota. The abundance of Basidiomycota increased in M24 (42.5%) and M31 (44.1%), but decreased again in M38 (26.7%). The pattern of relative abundance at the species level was similar to that at the genus level. The most abundant OTUs of each sampling period were *T. matsutake* (OTU 1, 94.0%) and *Cladosporium* sp. (OTU 7, 2.81%) in M03, *Pseudogymnoascus pannorum* (OTU 6, 28.5%) and *Oidiodendron echinulatum* (OTU 5, 21.5%) in M10, and *Wilcoxina mikolae* (OTU 2, 35.7%) in M17. After M24, the most abundant OTU was *Suillus granulatus* (OTU 3, 33.11% in M24; 25.19% in M31; 20.39% in M38) followed by *W. mikolae* (OTU 2, 11.84%) in M24, *S. luteus* in M31 (OTU 13, 7.37%), and *W. mikolae* (10.22%) in M38 (Figure 2C).

### Network Features and Correlation Within Fungal Community *P. densiflora* Seedlings

**FIGURE 3:**
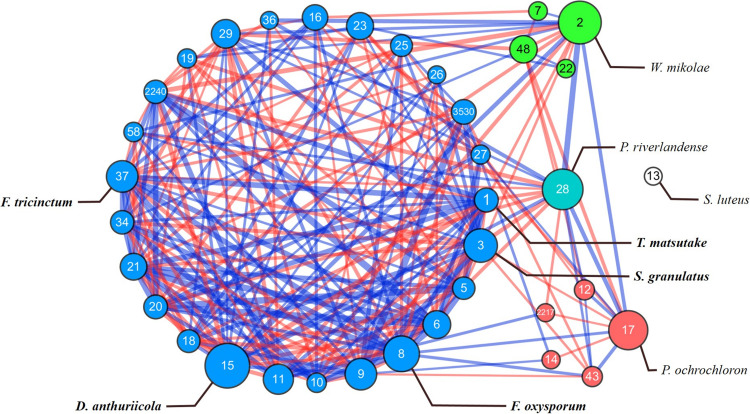
SparCC network map in OTU level in microbiome of *Pinus densiflora* seedlings. Each point represents a fungal species; edges indicate the relationship between species. Each clusters are shown in different colors, with isolated species are marked with white. The size of the nodes follows betweenness centrality scores. The width of the edges follows SparCC correlation coefficients (blue: positive correlation; red: negative correlation). Species with total relative abundance higher than 0.5% were chosen. Only statistically significant edges corresponding to correlations with a magnitude higher than 0.3 (*p* < 0.05) were drawn. Identity of each OTU is described in Table 2.

To identify the potential interactions among fungal species in *P. densiflora* seedlings, SparCC analysis was performed. *Tricholoma matsutake* (OTU 1) and 35 major OTUs with relative abundances >0.5%, accounting for 83.4% of total sequence reads, were clustered into four groups and one isolated OTU (Table 2). The network had a clustering coefficient of 0.589 and network centralization of 0.308 (Figure 3). Ten fungal OTUs showed significant positive correlations with *T. matsutake* (Supplementary Tables 1, 2). Most of these OTUs were saprotrophs or plant pathogens, with the exception of *O. echinulatum* (ericoid mycorrhiza). Based on our selection criteria, four OTUs were identified as keystone species during fungal succession of pine seedling roots: *Cylindrocarpon pauciseptatum* (OTU 15), *Suillus granulatus* (OTU 3), *Fusarium oxysporum* (OTU 8), and *Fusarium* sp. (OTU 37). All of the keystone taxa belonged to the same cluster. Among these keystone taxa, *Cylindrocarpon pauciseptatum* (OTU 15) and *S. granulatus* (OTU 3) showed significant negative correlation with *T. matsutake*, while *Fusarium oxysporum* (OTU 8) and *Fusarium* sp. (OTU 37) showed a positive correlation with *T. matsutake* (Supplementary Table 2).

**TABLE 2:** Major OTUs (relative abundance >0.5%) identity and node properties of the *Pinus densiflora* root seedling microbiome network.

ID	Species	Correlation with *T. matsutake*	Cluster	Betweenness centrality	Closeness centrality	Clustering coefficient	Degree	NCBI blast result	Accession No.	Identity	*E*-value
OTU 1	*Tricholoma matsutake*	–	1	0.017	0.667	0.669	17	*Tricholoma matsutake*	JF908729	100.00%	0.0
OTU 2	*Wilcoxina mikolae*	Negative	3	0.068	0.63	0.438	15	*Wilcoxina mikolae*	JQ310817.1	99.71%	2E-179
OTU 3	***Suillus granulatus***	**Negative**	1	0.043	0.694	0.544	19	*Suillus granulatus*	AY898617.1	98.59%	0.0
OTU 5	*Oidiodendron echinulatum*	Positive	1	0.013	0.618	0.692	14	*Oidiodendron echinulatum*	AF062791.1	100.00%	1E-166
OTU 6	*Pseudogymnoascus pannorum*	Positive	1	0.027	0.63	0.603	17	*Geomyces pannorum* (= *Pseudogymnoascus pannorum*)	JX131373.1	100.00%	9E-173
OTU 7	*Cladosporium* sp.	Neutral	3	0.002	0.459	0.333	3	*Cladosporium cladosporioides*	MK268136.1	100.00%	7E-174
OTU 8	***Fusarium oxysporum***	**Positive**	1	0.049	0.708	0.543	21	*Fusarium oxysporum*	MT453296.1	100.00%	2E-174
OTU 9	*Cadophora finlandica*	Negative	1	0.038	0.642	0.581	17	*Cadophora finlandica*	KT182905.1	97.82%	3E-153
OTU 10	*Leptosphaeria* sp.	Positive	1	0.004	0.586	0.821	13	*Leptosphaeria* sp.	JX238777	100.00%	4E-176
OTU 11	*Trichocladium* sp.	Positive	1	0.034	0.642	0.617	16	*Trichocladium* sp.*/ Humicola grisea* (= *Trichocladium griseum*)	MT348608.1/ MH860993.1	100.00%/ 100.00%	4E-176/ 4E-176
OTU 12	*Chaetomium* sp.	Neutral	2	0.006	0.507	0.5	5	*Chaetomium angustispirale/Humicola grisea*	MT453288.1/ MH860993.1	100.00%/ 100.00%	4E-176/ 4E-176
OTU 13	*Suillus luteus*	Neutral		0	0	0	0	*Suillus luteus*	KX213740.1	100.00%	0.0
OTU 14	*Tomentella* sp.	Neutral	2	0.001	0.459	0.333	3	*Tomentella tedersooi*	NR121359.1	95.09%	7E-180
OTU 15	***Cylindrocarpon pauciseptatum***	**Negative**	1	0.073	0.739	0.515	22	*Dactylonectria pauciseptata* (= *Cylindrocarpon pauciseptatum*)	MK602783.1	100.00%	0.0
OTU 16	*Ilyonectria* sp.	Neutral	1	0.021	0.531	0.778	10	*Ilyonectria liriodendri/Ilyonectria destructans*	MK602788.1	100.00%	0.0
OTU 17	*Penicillium ochrochloron*	Neutral	2	0.058	0.596	0.236	11	*Penicillium ochrochloron*	MK450704.1	100.00%	0.0
OTU 18	*Phialocephala fortinii*	Negative	1	0.013	0.618	0.714	15	*Phialocephala fortinii*	KF313097.1	100.00%	3E-167
OTU 19	*Dactylonectria* sp.	Neutral	1	0.004	0.531	0.778	9	*Dactylonectria torresensis*/*Dactylonectria alcacerensis*	MK602787/ MK602786	100.00%/ 100.00%	2E-180/ 2E-180
OTU 20	*Trichoderma viride*	Positive	1	0.016	0.63	0.657	15	*Trichoderma viride*	KU202217.1	100.00%	0.0
OTU 21	*Paraphaeosphaeria sporulosa*	Positive	1	0.024	0.68	0.643	19	*Paraphaeosphaeria sporulosa*	MT576023.1	100.00%	1E-176
OTU 22	*Xenochalara juniperi*	Neutral	3	0.002	0.442	0.333	3	*Xenochalara juniperi*	JX869564.1	100.00%	3E-172
OTU 23	Hyaloscyphaceae sp.	Neutral	1	0.025	0.567	0.472	9	Hyaloscyphaceae sp.	AB986450.1	97.61%	6E-160
OTU 25	Helotiales sp.	Neutral	1	0.011	0.515	0.778	9	Helotiales sp.	LC218319.1	100.00%	4E-166
OTU 26	*Trichoderma* sp.	Neutral	1	0	0.515	0.952	7	*Trichoderma* sp.	MK871291.1	100.00%	0.0
OTU 27	*Oidiodendron rhodogenum*	Neutral	1	0.004	0.531	0.714	7	*Oidiodendron rhodogenum*	AF062803.1	100.00%	4E-166
OTU 28	*Penicillium* sp.	Negative	4	0.062	0.654	0.442	16	*Penicillium* sp.	MK450684.1	100.00%	0.0
OTU 29	*Oidiodendron* sp.	Neutral	1	0.03	0.654	0.6	16	*Oidiodendron tenuissimum/Oidiodendron griseum*	MH864345.1/ AF062797.1	99.69%/ 99.69%	4E-166/ 6E-165
OTU 34	*Knufia* sp.	Positive	1	0.015	0.618	0.725	14	*Knufia* sp.	KX610444.1	98.79%	8E-164
OTU 36	*Entrophospora* sp.	Neutral	1	0	0.515	1	6	*Entrophospora* sp.	AY035666.1	99.12%	3E-172
OTU 37	***Fusarium* sp.**	**Positive**	1	0.038	0.708	0.59	21	*Fusarium acuminatum/Fusarium tricinctum*	MT294407.1/ MT453281.1	100.00%/ 100.00%	0.0/ 0.0
OTU 43	*Talaromyces* sp.	Neutral	2	0.007	0.531	0.524	7	*Talaromyces amestolkiae*	MN511323.1	100.00%	1E-177
OTU 48	*Exophiala* sp.	Neutral	3	0.026	0.557	0.357	8	*Exophiala* sp.	MF619956.1	100.00%	0.0
OTU 58	*Sebacina* sp.	Negative	1	0.005	0.586	0.711	10	*Sebacina* sp.	KY271862.1	96.61%	3E-178
OTU 2217	*Corynascella inaequalis*	Neutral	2	0.001	0.453	0.667	3	*Corynascella inaequalis*	MT453282.1	99.41%	1E-172
OTU 2240	*Pseudogymnoascus pannorum*	Positive	1	0.015	0.642	0.667	16	*Pseudogymnoascus pannorum*	MH854616.1	99.70%	4E-171
OTU 3530	*Cadophora finlandica*	Neutral	1	0.019	0.596	0.679	13	*Cadophora finlandica*	DQ069045.1	99.03%	3E-153

*OTUs in bold font indicate keystone taxa.*

## Discussion

### Change of Fungal Communities in Pine Seedlings After Transplantation

The root fungal communities significantly changed through seedling development. Our results showed that root colonization of *T. matsutake* dramatically decreased after being transplanted to the greenhouse, and they were replaced by other fungi. As expected, alpha diversity increased when seedlings were transplanted from a controlled environment to a more natural, open environment. After transplantation, fast-growing Ascomycota dominated, and then were replaced by early-stage ectomycorrhizal fungi. Previous studies looked at the mycorrhizal succession in pine seedlings (Peay et al., 2011; Herzog et al., 2019; Rudawska et al., 2019), and we found that the shift of root associated fungi in our study followed the general trend, despite being inoculated with *T. matsutake*.

After transplantation to the greenhouse, Ascomycota species became dominant in seedling roots. In M10, most of the dominant OTUs were saprotrophs or pathotrophs, such as *Pseudogymnoascus* and *Fusarium*, with one exception being *Oidiodendron echinulatum*, an ericoid mycorrhizal fungus. In M17, the abundance of symbiotrophs (e.g., *Wilcoxina mikolae*) increased, while pathotrophs decreased. The presence of saprotrophic fungi is commonly reported in roots (Vasiliauskas et al., 2007; Tedersoo and Smith, 2013; Lee et al., 2015; Rincón et al., 2015; Smith et al., 2017), so we believe their presence in our study is not a result of inappropriate sterilization of roots. Among the saprotroph species identified in our study, *Pseudogymnoascus pannorum* is widely distributed in the soil and adapted to nutrient poor environments (Minnis and Lindner, 2013; Chaturvedi et al., 2018). Previous studies of pine seedling roots also discovered the presence of *Pseudogymnoascus* species (Menkis and Vasaitis, 2011; Moler and Aho, 2018). Other taxa, like *Oidiodendron* and *Wilcoxina*, are well known species that are common in early successional or disturbed ecosystems (Berch et al., 2006; Lee et al., 2012; Lee and Eom, 2013; Rudawska et al., 2019). As these species were absent in samples from M03, and both taxa found in our study are expected to have been dispersed by wind (Horton, 2017). A noteworthy result is the high abundance of *Fusarium* in M10–M17 samples. Usually, *Fusarium* is considered a plant pathogen (Gordon, 2017), but *Fusarium* species have also been found as endophytes of a wide range of wild plants (Kuldau and Yates, 2000; Min et al., 2014). For example, growth-enhancement or pathogen-resistance conferred by non-pathogenic *Fusarium* species were widely reported (Forsyth et al., 2006; Waweru et al., 2014). Their role is uncertain in our study, and further study would be needed to understand *Fusarium*’s role in roots of pine seedlings.

After M24, we witnessed an increase in proportion of ectomycorrhizal (*Suillus* and *Tomentella*) and endophytic fungi (*Cadophora* and *Phialocephala*), which are considered common fungi in an early successional stage (Colpaert et al., 1996; Berch et al., 2006; Sim and Eom, 2009; Lee et al., 2012; Lee and Eom, 2013; Lee and Koo, 2016). In particular, *Suillus* species are known to be important in the establishment of pine seedlings (Hayward et al., 2015). *Suillus* species might be more competitive than other mycorrhizal fungi found in first year, such as *Wilcoxina*. *Wilcoxina* is known as a weak competitor ectomycorrhizal fungus that prospers only in absence of competitor ectomycorrhizal fungi (Danielson and Prudel, 1990). *Suillus* species are known to form ectomycorrhiza with pine trees that span a large area, thanks to long distance dispersal of spores combined with large sporocarps and a high volume of spore production and (Peay et al., 2012; Horton, 2017). Other species, such as *Cadophora* and *Tomentella*, are considered common fungi of pine seedlings in an early successional stage or disturbed areas (Colpaert et al., 1996; Berch et al., 2006; Sim and Eom, 2009; Lee et al., 2012; Lee and Eom, 2013; Lee and Koo, 2016).

While *T. matsutake* was still found in several *P. densiflora* seedlings, its frequency and abundance steadily decreased over time after transplantation. Although the priority effect in ectomycorrhiza was reported in previous studies (Kennedy and Bruns, 2005; Kennedy et al., 2009; Fukumi, 2015), it did not apply to *T. matsutake* in our study. As *T. matsutake* is usually known to form symbiotic relationship with mature pine trees in the field (Wang et al., 2017), our results suggest that the symbiosis between *T. matsutake* and young seedlings is not sustainable outside of sterile environment without proper support. We suggest that this is due to a slow growth rate and higher carbon demand of *T. matsutake* as a late-stage ectomycorrhizal fungus (Smith and Read, 2010).

### Network Analysis and Keystone Taxa

Microbial network analysis has been used to visualize taxa with a strong effect on network structure, or highly connected taxa in various environments (Barberán et al., 2012; Gilbert et al., 2012; Agler et al., 2016). We constructed a network of 35 fungal OTUs that were abundant during pine seedling growth with SparCC correlations. Interaction and network formation between functionally diverse fungi were previously reported (Toju et al., 2016), and our results were similar; a combination of functionally different OTUs were observed in each cluster in our network (Supplementary Table 1).

Among the 35 major OTUs, 10 OTUs were found to have positive correlations with *T. matsutake*, despite the abundance of *T. matsutake* decreasing after transplantation (Table 1, Supplementary Tables 1, 2). Among these positively correlated OTUs, *Fusarium*, *Trichoderma*, and/or *Oidiodendron* might improve survival of *T. matsutake* in our environment. While competition between ectomycorrhiza and other microfungi are common in soil (Leake et al., 2003), several studies reported growth promotion of ectomycorrhizal fungi by microfungi isolated from soil (Ogawa, 1976; Oh et al., 2018). For instance, *Trichoderma* and *Oidiodendron* species were exclusively isolated from the *T. matsutake* fruiting zone of *P. densiflora* forests (Ogawa, 1977; Oh et al., 2018, 2019), and high abundance of *F. oxysporum* was reported in *Tuber magnatum*-productive areas (Mello et al., 2010). Likewise, we found that ectomycorrhizal fungi, such as *Suillus* were less abundant in root samples with *T. matsutake* than those without *T. matsutake*. *Trichoderma* might help survival of *T. matsutake* by promoting plant growth, root branching, and development (Guzmán-Guzmán et al., 2019), or by inhibiting the growth of other ectomycorrhiza as reported between *Trichoderma viride* and *Suillus bovinus* in soil environment (Mucha et al., 2008; Sabella et al., 2015; Oh et al., 2018, 2019).

Four OTUs were identified as keystone taxa: *S. granulatus*, *C. pauciseptatum*, *Fusarium* sp. (OTU 37), and *Fusarium oxysporum* (Table 2). Keystone taxa are taxa highly connected to other network members and play important roles in the microbiome (Banerjee et al., 2018), and they are required to understand an ecosystem’s response to disturbance (Stinson et al., 2006). Of the four keystone taxa, *S. granulatus* and *C. pauciseptatum* showed a significantly negative correlation with *T. matsutake*, while *F. oxysporum* and *Fusarium* sp. (OTU 37) showed a significantly positive correlation with *T. matsutake*. As *Suillus granulatus* is a strong competitor and crucial in the establishment of pine seedlings (Dickie et al., 2010; Kohout et al., 2011; Hayward et al., 2015; Urcelay et al., 2017; Policelli et al., 2019), its negative relationship with *T. matsutake* is as expected. However, the significant correlation between *T. matsutake* and *C. pauciseptatum* or *Fusarium* species was interesting, as *C. pauciseptatum* and *Fusarium* species are known as soil saprotrophs or plant pathogens. The presence of *C. pauciseptatum* was reported in *Pinus sylvestris* (Menkis and Vasaitis, 2011), the relationship between *C. pauciseptatum* and *P. densiflora* is still unknown. It is possible that *C. pauciseptatum* indirectly influenced microbiome by affecting quality of pine seedlings (Agler et al., 2016). *Fusarium oxysporum* and *F. trincintum* are known as plant pathogens or mutualistic endophytes (Kuldau and Yates, 2000; Forsyth et al., 2006; Vu et al., 2006; Michielse and Rep, 2009; Min et al., 2014; Waweru et al., 2014; Vasundhara et al., 2016). While we do not understand their exact function in this study, both endophyte and plant pathogen might influence on root microbiome by positive or negative effects (Van Der Heijden et al., 2008).

## Conclusion

We have documented the change in fungal community composition in pine seedlings after the *T. matsutake* inoculation, and introduced a SparCC analysis to predict the cross-fungi associations from NGS data. The root microbiome drastically changed at alpha- and beta-diversity levels after transplantation. Temporal succession of the mycorrhizal community suggests a weak priority effect as *T. matsutake* was rapidly replaced by *W. mikolae*, *S. granulatus*, and other fungi. While most of the major fungal OTUs showed negative or neutral correlation with *T. matsutake*, some of them showed a positive relationship. Fungi that had a positive correlation with *T. matsutake* were mostly known as saprotrophs or plant pathogens. In addition, we found four keystone species during microbiome succession that might play an important role in microbiome composition in pine seedlings. A further study is needed to verify the effect of fungi that have positive correlations with *T. matsutake* in an artificial cultivation of ectomycorrhizal fungi.

## Data Availability Statement

The datasets generated for this study can be found in the NCBI Sequence Read Archive (SRA) under BioProject ID PRJNA638021 (https://www.ncbi.nlm.nih.gov/bioproject/PRJN A638021/) and also available on request to the corresponding author.

## Author Contributions

S-YO, MP, and YL contributed to conceiving and designing the experiments. KP and S-YO performed the experiments and analyzed the data. KP, S-YO, and SY wrote the manuscript with revisions from JF, MP, and YL. All authors contributed to the article and approved the submitted version.

## Conflict of Interest

The authors declare that the research was conducted in the absence of any commercial or financial relationships that could be construed as a potential conflict of interest.
