# Supplementary material for: Successional Change of the Fungal Microbiome Pine Seedling Roots Inoculated With Tricholoma matsutake
Source: Front Microbiol. 2020 Sep 25;11:574146. doi: 10.3389/fmicb.2020.574146 (PMC7545793; doi:10.3389/fmicb.2020.574146)
Supplement: Supplementary file 1 [file Data_Sheet_1.docx]

**Supplementary Table 1. Results from the post-hoc PERMANOVA (Adonis) among sampling groups**

| **Pairs** | **Df** | **SumsOfSqs** | **F.Model** | **R^2^** | **p.value** | **p.adjusted** |
| --- | --- | --- | --- | --- | --- | --- |
| M10 vs M17 | 1 | 3.008 | 19.812 | 0.398 | 0.001 | 0.01 |
| M10 vs M24 | 1 | 3.827 | 20.836 | 0.410 | 0.001 | 0.01 |
| M10 vs M31 | 1 | 3.473 | 16.451 | 0.354 | 0.001 | 0.01 |
| M10 vs M38 | 1 | 3.531 | 15.712 | 0.344 | 0.001 | 0.01 |
| M17 vs M24 | 1 | 2.599 | 16.825 | 0.359 | 0.001 | 0.01 |
| M17 vs M31 | 1 | 3.142 | 17.270 | 0.365 | 0.001 | 0.01 |
| M17 vs M38 | 1 | 2.456 | 12.560 | 0.295 | 0.001 | 0.01 |
| M24 vs M31 | 1 | 0.609 | 2.851 | 0.087 | 0.008 | 0.08 |
| M24 vs M38 | 1 | 0.823 | 3.619 | 0.108 | 0.004 | 0.04 |
| M31 vs M38 | 1 | 0.883 | 3.464 | 0.104 | 0.001 | 0.01 |

**Supplementary Table 2. Edge properties of the *Pinus densiflora* seedling microbiome network.** Identity of OTUs are available in Table 2.

| OTU(A) | cluster A | OTU(B) | cluster B | SparCC correlation (A-B) | p-value | Relationship |
| --- | --- | --- | --- | --- | --- | --- |
| OTU 1 | 1 | OTU 2 | 3 | -0.365516193 | 0 | negative |
| OTU 1 | 1 | OTU 3 | 1 | -0.341403498 | 0 | negative |
| OTU 1 | 1 | OTU 5 | 1 | 0.51606342 | 0 | positive |
| OTU 1 | 1 | OTU 6 | 1 | 0.690188234 | 0 | positive |
| OTU 1 | 1 | OTU 8 | 1 | 0.47354543 | 0 | positive |
| OTU 1 | 1 | OTU 9 | 1 | -0.363899197 | 0 | negative |
| OTU 1 | 1 | OTU 10 | 1 | 0.331624281 | 0 | positive |
| OTU 1 | 1 | OTU 11 | 1 | 0.469717136 | 0 | positive |
| OTU 1 | 1 | OTU 15 | 1 | -0.585373116 | 0 | negative |
| OTU 1 | 1 | OTU 18 | 1 | -0.409854635 | 0 | negative |
| OTU 1 | 1 | OTU 20 | 1 | 0.596388608 | 0 | positive |
| OTU 1 | 1 | OTU 21 | 1 | 0.643233824 | 0 | positive |
| OTU 1 | 1 | OTU 28 | 4 | -0.447206026 | 0 | negative |
| OTU 1 | 1 | OTU 34 | 1 | 0.384218538 | 0 | positive |
| OTU 1 | 1 | OTU 37 | 1 | 0.659680296 | 0 | positive |
| OTU 1 | 1 | OTU 58 | 1 | -0.363613059 | 0 | negative |
| OTU 1 | 1 | OTU 2240 | 1 | 0.599278922 | 0 | positive |
| OTU 2 | 3 | OTU 5 | 1 | -0.344683473 | 0 | negative |
| OTU 2 | 3 | OTU 7 | 3 | 0.312423704 | 0 | positive |
| OTU 2 | 3 | OTU 11 | 1 | -0.484490731 | 0 | negative |
| OTU 2 | 3 | OTU 15 | 1 | 0.524009708 | 0 | positive |
| OTU 2 | 3 | OTU 17 | 2 | 0.452448064 | 0 | positive |
| OTU 2 | 3 | OTU 19 | 1 | 0.323500753 | 0 | positive |
| OTU 2 | 3 | OTU 20 | 1 | -0.435635335 | 0 | negative |
| OTU 2 | 3 | OTU 22 | 3 | -0.312175235 | 0 | negative |
| OTU 2 | 3 | OTU 28 | 4 | 0.673481742 | 0 | positive |
| OTU 2 | 3 | OTU 29 | 1 | 0.387557133 | 0 | positive |
| OTU 2 | 3 | OTU 36 | 1 | 0.506577952 | 0 | positive |
| OTU 2 | 3 | OTU 48 | 3 | -0.399953374 | 0 | negative |
| OTU 2 | 3 | OTU 58 | 1 | 0.379399931 | 0 | positive |
| OTU 2 | 3 | OTU 2240 | 1 | -0.570834978 | 0 | negative |
| OTU 3 | 1 | OTU 6 | 1 | -0.404025436 | 0 | negative |
| OTU 3 | 1 | OTU 8 | 1 | -0.581953866 | 0 | negative |
| OTU 3 | 1 | OTU 9 | 1 | 0.678473928 | 0 | positive |
| OTU 3 | 1 | OTU 10 | 1 | -0.499255338 | 0 | negative |
| OTU 3 | 1 | OTU 15 | 1 | 0.415857718 | 0 | positive |
| OTU 3 | 1 | OTU 16 | 1 | -0.300757606 | 0 | negative |
| OTU 3 | 1 | OTU 17 | 2 | -0.313098685 | 0 | negative |
| OTU 3 | 1 | OTU 18 | 1 | 0.514949078 | 0 | positive |
| OTU 3 | 1 | OTU 21 | 1 | -0.432259508 | 0 | negative |
| OTU 3 | 1 | OTU 23 | 1 | 0.323562773 | 0 | positive |
| OTU 3 | 1 | OTU 25 | 1 | 0.406624054 | 0 | positive |
| OTU 3 | 1 | OTU 26 | 1 | 0.455237643 | 0 | positive |
| OTU 3 | 1 | OTU 29 | 1 | 0.579505276 | 0 | positive |
| OTU 3 | 1 | OTU 34 | 1 | -0.482016074 | 0 | negative |
| OTU 3 | 1 | OTU 37 | 1 | -0.489134145 | 0 | negative |
| OTU 3 | 1 | OTU 43 | 2 | -0.338608418 | 0.01 | negative |
| OTU 3 | 1 | OTU 58 | 1 | 0.314481833 | 0 | positive |
| OTU 3 | 1 | OTU 3530 | 1 | 0.579780005 | 0 | positive |
| OTU 5 | 1 | OTU 6 | 1 | 0.768644257 | 0 | positive |
| OTU 5 | 1 | OTU 8 | 1 | 0.311998922 | 0.01 | positive |
| OTU 5 | 1 | OTU 11 | 1 | 0.404422475 | 0 | positive |
| OTU 5 | 1 | OTU 15 | 1 | -0.455373126 | 0 | negative |
| OTU 5 | 1 | OTU 19 | 1 | -0.414663311 | 0 | negative |
| OTU 5 | 1 | OTU 20 | 1 | 0.75069351 | 0 | positive |
| OTU 5 | 1 | OTU 21 | 1 | 0.50138122 | 0 | positive |
| OTU 5 | 1 | OTU 27 | 1 | 0.530503112 | 0 | positive |
| OTU 5 | 1 | OTU 28 | 4 | -0.448828348 | 0 | negative |
| OTU 5 | 1 | OTU 36 | 1 | -0.300998618 | 0 | negative |
| OTU 5 | 1 | OTU 37 | 1 | 0.332697965 | 0 | positive |
| OTU 5 | 1 | OTU 2240 | 1 | 0.637885909 | 0 | positive |
| OTU 6 | 1 | OTU 8 | 1 | 0.644212762 | 0 | positive |
| OTU 6 | 1 | OTU 10 | 1 | 0.400991845 | 0 | positive |
| OTU 6 | 1 | OTU 11 | 1 | 0.611400365 | 0 | positive |
| OTU 6 | 1 | OTU 15 | 1 | -0.510298621 | 0 | negative |
| OTU 6 | 1 | OTU 18 | 1 | -0.40422037 | 0 | negative |
| OTU 6 | 1 | OTU 19 | 1 | -0.301145668 | 0 | negative |
| OTU 6 | 1 | OTU 20 | 1 | 0.783384601 | 0 | positive |
| OTU 6 | 1 | OTU 21 | 1 | 0.743398013 | 0 | positive |
| OTU 6 | 1 | OTU 23 | 1 | -0.354526756 | 0 | negative |
| OTU 6 | 1 | OTU 26 | 1 | -0.322451545 | 0 | negative |
| OTU 6 | 1 | OTU 27 | 1 | 0.514174753 | 0 | positive |
| OTU 6 | 1 | OTU 34 | 1 | 0.402420387 | 0 | positive |
| OTU 6 | 1 | OTU 37 | 1 | 0.624910811 | 0 | positive |
| OTU 6 | 1 | OTU 2240 | 1 | 0.783254363 | 0 | positive |
| OTU 7 | 3 | OTU 16 | 1 | -0.353983143 | 0.01 | negative |
| OTU 7 | 3 | OTU 48 | 3 | -0.342732535 | 0 | negative |
| OTU 8 | 1 | OTU 9 | 1 | -0.497362744 | 0 | negative |
| OTU 8 | 1 | OTU 10 | 1 | 0.675958117 | 0 | positive |
| OTU 8 | 1 | OTU 11 | 1 | 0.422550963 | 0 | positive |
| OTU 8 | 1 | OTU 15 | 1 | -0.384224845 | 0 | negative |
| OTU 8 | 1 | OTU 16 | 1 | 0.401201677 | 0 | positive |
| OTU 8 | 1 | OTU 17 | 2 | 0.377995668 | 0 | positive |
| OTU 8 | 1 | OTU 18 | 1 | -0.493092113 | 0 | negative |
| OTU 8 | 1 | OTU 20 | 1 | 0.415490211 | 0 | positive |
| OTU 8 | 1 | OTU 21 | 1 | 0.719252957 | 0 | positive |
| OTU 8 | 1 | OTU 25 | 1 | -0.407598763 | 0 | negative |
| OTU 8 | 1 | OTU 26 | 1 | -0.310287733 | 0 | negative |
| OTU 8 | 1 | OTU 29 | 1 | -0.466064441 | 0 | negative |
| OTU 8 | 1 | OTU 34 | 1 | 0.580593334 | 0 | positive |
| OTU 8 | 1 | OTU 37 | 1 | 0.637530565 | 0 | positive |
| OTU 8 | 1 | OTU 43 | 2 | 0.39738259 | 0 | positive |
| OTU 8 | 1 | OTU 2240 | 1 | 0.402066103 | 0 | positive |
| OTU 8 | 1 | OTU 3530 | 1 | -0.472668847 | 0 | negative |
| OTU 9 | 1 | OTU 10 | 1 | -0.483192638 | 0 | negative |
| OTU 9 | 1 | OTU 14 | 2 | 0.311021354 | 0.01 | positive |
| OTU 9 | 1 | OTU 15 | 1 | 0.415818751 | 0 | positive |
| OTU 9 | 1 | OTU 16 | 1 | -0.313397092 | 0.02 | negative |
| OTU 9 | 1 | OTU 18 | 1 | 0.451597811 | 0 | positive |
| OTU 9 | 1 | OTU 21 | 1 | -0.405623994 | 0 | negative |
| OTU 9 | 1 | OTU 25 | 1 | 0.513739047 | 0 | positive |
| OTU 9 | 1 | OTU 26 | 1 | 0.334489958 | 0 | positive |
| OTU 9 | 1 | OTU 29 | 1 | 0.447225694 | 0 | positive |
| OTU 9 | 1 | OTU 34 | 1 | -0.364804333 | 0 | negative |
| OTU 9 | 1 | OTU 37 | 1 | -0.515518182 | 0 | negative |
| OTU 9 | 1 | OTU 43 | 2 | -0.301276319 | 0 | negative |
| OTU 9 | 1 | OTU 58 | 1 | 0.35943485 | 0 | positive |
| OTU 9 | 1 | OTU 3530 | 1 | 0.799836413 | 0 | positive |
| OTU 10 | 1 | OTU 16 | 1 | 0.39790165 | 0 | positive |
| OTU 10 | 1 | OTU 18 | 1 | -0.32242917 | 0 | negative |
| OTU 10 | 1 | OTU 21 | 1 | 0.517122501 | 0 | positive |
| OTU 10 | 1 | OTU 25 | 1 | -0.325751928 | 0 | negative |
| OTU 10 | 1 | OTU 29 | 1 | -0.422775435 | 0 | negative |
| OTU 10 | 1 | OTU 34 | 1 | 0.666808675 | 0 | positive |
| OTU 10 | 1 | OTU 37 | 1 | 0.559718356 | 0 | positive |
| OTU 10 | 1 | OTU 3530 | 1 | -0.447226058 | 0 | negative |
| OTU 11 | 1 | OTU 15 | 1 | -0.496224227 | 0 | negative |
| OTU 11 | 1 | OTU 19 | 1 | -0.369649277 | 0.01 | negative |
| OTU 11 | 1 | OTU 20 | 1 | 0.504217724 | 0 | positive |
| OTU 11 | 1 | OTU 21 | 1 | 0.481837447 | 0 | positive |
| OTU 11 | 1 | OTU 28 | 4 | -0.5158019 | 0 | negative |
| OTU 11 | 1 | OTU 29 | 1 | -0.516696349 | 0 | negative |
| OTU 11 | 1 | OTU 36 | 1 | -0.361409664 | 0.01 | negative |
| OTU 11 | 1 | OTU 37 | 1 | 0.40955612 | 0.01 | positive |
| OTU 11 | 1 | OTU 58 | 1 | -0.362487803 | 0 | negative |
| OTU 11 | 1 | OTU 2217 | 2 | 0.384608657 | 0.01 | positive |
| OTU 11 | 1 | OTU 2240 | 1 | 0.638342753 | 0 | positive |
| OTU 12 | 2 | OTU 17 | 2 | 0.402239911 | 0 | positive |
| OTU 12 | 2 | OTU 25 | 1 | -0.305925904 | 0 | negative |
| OTU 12 | 2 | OTU 28 | 4 | 0.321980036 | 0 | positive |
| OTU 12 | 2 | OTU 43 | 2 | 0.304466782 | 0 | positive |
| OTU 12 | 2 | OTU 48 | 3 | -0.314929498 | 0.01 | negative |
| OTU 13 | 0 | OTU 13 | 0 | 0 | 0 | none |
| OTU 14 | 2 | OTU 17 | 2 | -0.320150978 | 0 | negative |
| OTU 14 | 2 | OTU 3530 | 1 | 0.363073357 | 0 | positive |
| OTU 15 | 1 | OTU 18 | 1 | 0.438033267 | 0 | positive |
| OTU 15 | 1 | OTU 19 | 1 | 0.4033535 | 0 | positive |
| OTU 15 | 1 | OTU 20 | 1 | -0.609534856 | 0 | negative |
| OTU 15 | 1 | OTU 21 | 1 | -0.437302917 | 0 | negative |
| OTU 15 | 1 | OTU 23 | 1 | 0.325877431 | 0.01 | positive |
| OTU 15 | 1 | OTU 26 | 1 | 0.34044479 | 0 | positive |
| OTU 15 | 1 | OTU 27 | 1 | -0.306802316 | 0.03 | negative |
| OTU 15 | 1 | OTU 28 | 4 | 0.436697158 | 0 | positive |
| OTU 15 | 1 | OTU 29 | 1 | 0.313257007 | 0 | positive |
| OTU 15 | 1 | OTU 34 | 1 | -0.386944546 | 0 | negative |
| OTU 15 | 1 | OTU 36 | 1 | 0.350911361 | 0 | positive |
| OTU 15 | 1 | OTU 37 | 1 | -0.509789017 | 0 | negative |
| OTU 15 | 1 | OTU 2240 | 1 | -0.556504962 | 0 | negative |
| OTU 15 | 1 | OTU 3530 | 1 | 0.312390833 | 0.01 | positive |
| OTU 16 | 1 | OTU 21 | 1 | 0.390137669 | 0 | positive |
| OTU 16 | 1 | OTU 29 | 1 | -0.344618368 | 0.03 | negative |
| OTU 16 | 1 | OTU 34 | 1 | 0.370431162 | 0.01 | positive |
| OTU 16 | 1 | OTU 37 | 1 | 0.439842727 | 0 | positive |
| OTU 16 | 1 | OTU 3530 | 1 | -0.348805251 | 0 | negative |
| OTU 17 | 2 | OTU 23 | 1 | -0.393046059 | 0 | negative |
| OTU 17 | 2 | OTU 27 | 1 | 0.303755567 | 0.01 | positive |
| OTU 17 | 2 | OTU 28 | 4 | 0.512645913 | 0 | positive |
| OTU 17 | 2 | OTU 43 | 2 | 0.4413373 | 0 | positive |
| OTU 17 | 2 | OTU 48 | 3 | -0.444977587 | 0 | negative |
| OTU 17 | 2 | OTU 2217 | 2 | -0.308504548 | 0 | negative |
| OTU 18 | 1 | OTU 21 | 1 | -0.469952291 | 0 | negative |
| OTU 18 | 1 | OTU 23 | 1 | 0.448741357 | 0 | positive |
| OTU 18 | 1 | OTU 25 | 1 | 0.439751002 | 0 | positive |
| OTU 18 | 1 | OTU 26 | 1 | 0.327490807 | 0 | positive |
| OTU 18 | 1 | OTU 29 | 1 | 0.348296706 | 0 | positive |
| OTU 18 | 1 | OTU 34 | 1 | -0.329231556 | 0 | negative |
| OTU 18 | 1 | OTU 37 | 1 | -0.478569182 | 0 | negative |
| OTU 18 | 1 | OTU 3530 | 1 | 0.32004942 | 0.01 | positive |
| OTU 19 | 1 | OTU 20 | 1 | -0.36326458 | 0.01 | negative |
| OTU 19 | 1 | OTU 23 | 1 | 0.38031654 | 0 | positive |
| OTU 19 | 1 | OTU 27 | 1 | -0.482630881 | 0 | negative |
| OTU 19 | 1 | OTU 2240 | 1 | -0.339075726 | 0 | negative |
| OTU 20 | 1 | OTU 21 | 1 | 0.638973914 | 0 | positive |
| OTU 20 | 1 | OTU 23 | 1 | -0.424798873 | 0 | negative |
| OTU 20 | 1 | OTU 27 | 1 | 0.43740739 | 0 | positive |
| OTU 20 | 1 | OTU 28 | 4 | -0.434450365 | 0 | negative |
| OTU 20 | 1 | OTU 37 | 1 | 0.443815341 | 0 | positive |
| OTU 20 | 1 | OTU 58 | 1 | -0.301292269 | 0 | negative |
| OTU 20 | 1 | OTU 2240 | 1 | 0.649049055 | 0 | positive |
| OTU 21 | 1 | OTU 25 | 1 | -0.419782385 | 0 | negative |
| OTU 21 | 1 | OTU 29 | 1 | -0.304915312 | 0.01 | negative |
| OTU 21 | 1 | OTU 34 | 1 | 0.435235217 | 0 | positive |
| OTU 21 | 1 | OTU 37 | 1 | 0.639037382 | 0 | positive |
| OTU 21 | 1 | OTU 58 | 1 | -0.321980871 | 0 | negative |
| OTU 21 | 1 | OTU 2240 | 1 | 0.542119452 | 0 | positive |
| OTU 21 | 1 | OTU 3530 | 1 | -0.381995144 | 0 | negative |
| OTU 22 | 3 | OTU 23 | 1 | 0.343483106 | 0 | positive |
| OTU 22 | 3 | OTU 48 | 3 | 0.316723468 | 0 | positive |
| OTU 23 | 1 | OTU 27 | 1 | -0.438554757 | 0 | negative |
| OTU 25 | 1 | OTU 37 | 1 | -0.314503571 | 0 | negative |
| OTU 25 | 1 | OTU 3530 | 1 | 0.360251857 | 0 | positive |
| OTU 26 | 1 | OTU 37 | 1 | -0.320071002 | 0 | negative |
| OTU 28 | 4 | OTU 29 | 1 | 0.336886826 | 0 | positive |
| OTU 28 | 4 | OTU 36 | 1 | 0.477400834 | 0 | positive |
| OTU 28 | 4 | OTU 37 | 1 | -0.320643448 | 0.01 | negative |
| OTU 28 | 4 | OTU 43 | 2 | 0.349260392 | 0 | positive |
| OTU 28 | 4 | OTU 48 | 3 | -0.363769533 | 0.01 | negative |
| OTU 28 | 4 | OTU 58 | 1 | 0.351340446 | 0 | positive |
| OTU 28 | 4 | OTU 2217 | 2 | -0.352025379 | 0 | negative |
| OTU 28 | 4 | OTU 2240 | 1 | -0.524482211 | 0 | negative |
| OTU 29 | 1 | OTU 34 | 1 | -0.494518024 | 0 | negative |
| OTU 29 | 1 | OTU 37 | 1 | -0.494079791 | 0 | negative |
| OTU 29 | 1 | OTU 48 | 3 | -0.46731176 | 0 | negative |
| OTU 29 | 1 | OTU 2240 | 1 | -0.39267545 | 0 | negative |
| OTU 29 | 1 | OTU 3530 | 1 | 0.353579325 | 0 | positive |
| OTU 34 | 1 | OTU 37 | 1 | 0.564472973 | 0 | positive |
| OTU 34 | 1 | OTU 48 | 3 | 0.332749108 | 0.01 | positive |
| OTU 34 | 1 | OTU 2240 | 1 | 0.308918109 | 0 | positive |
| OTU 36 | 1 | OTU 2240 | 1 | -0.41089654 | 0.01 | negative |
| OTU 37 | 1 | OTU 58 | 1 | -0.310981376 | 0 | negative |
| OTU 37 | 1 | OTU 2240 | 1 | 0.54003854 | 0 | positive |
| OTU 37 | 1 | OTU 3530 | 1 | -0.397898098 | 0 | negative |
| OTU 43 | 2 | OTU 3530 | 1 | -0.3530239 | 0 | negative |
| OTU 58 | 3 | OTU 2240 | 1 | -0.324367439 | 0 | negative |
